# Necroptosis-associated classification combined with tumor microenvironment characteristic analysis of cutaneous melanoma

**DOI:** 10.1038/s41598-022-12676-6

**Published:** 2022-05-24

**Authors:** Gang Hu, Yan Jiang, Jianying Ma, Hui Zhan

**Affiliations:** 1 Department of Breast Surgery, Thyroid Surgery, Huangshi Central Hospital of Edong Healthcare Group, Affiliated Hospital of Hubei Polytechnic University, Huangshi, Hubei China; 2 Department of Nosocomial Infection Management, Huangshi Central Hospital of Edong Healthcare Group, Affiliated Hospital of Hubei Polytechnic University, Huangshi, Hubei China; 3 Department of Dermatology, Huangshi Central Hospital (Pu Ai Hospital) of Edong Healthcare Group, Affiliated Hospital of Hubei Polytechnic University, No. 141, Tianjin Road, Huangshi, 435000 Hubei China

**Keywords:** Cancer microenvironment, Skin cancer, Tumour biomarkers, Tumour heterogeneity, Tumour immunology, Necroptosis, Biomarkers, Risk factors

## Abstract

Necroptosis is a mode of programmed cell death that overcomes apoptotic resistance. The accurate prognosis of cutaneous melanoma is complicated to predict due to tumor heterogeneity. Necroptosis contributes to the regulation of oncogenesis and cancer immunity. We comprehensively investigated different necroptosis patterns by the non-negative matrix factorization (NMF) clustering analysis and explored the relationships among necroptosis patterns, infiltered immune cells, and tumor microenvironment (TME) scores. Two different necroptosis patterns were identified, and the two clusters could predict prognosis and immune landscape. A four-gene signature was successfully constructed and validated its predictive capability of overall survival (OS) in cutaneous melanoma patients. The prognostic value of the signature was further enhanced by incorporating other independent prognostic factors such as age and clinicopathological stages in a nomogram-based prediction model. Patients with lower risk scores tended to have better OS, higher TME score, immune checkpoints, immunophenoscore (IPS), and lower Tumor Immune Dysfunction and Exclusion (TIDE), which indicated better responses to immunotherapy. In addition, the pigmentation score of the high-risk group was visibly higher than those of the low-risk group. In conclusion, the necroptosis-related signature indicated favorable predictive performance in cutaneous melanoma patients, which provides guidance for immunotherapy and provide novel insights into precision medicine.

## Introduction

Cutaneous melanoma is the most malignant skin cancer with great potential to develop metastases. It accounts for less than 5% of the overall cutaneous malignancies but contributes to almost all skin cancer deaths^1^. The incidence of primary cutaneous melanoma continues to increase each year^2^. Despite advances in the treatment of metastatic melanoma and prevention of metastatic recurrence, clinical prognosis remains poor^3^. Therefore, developing cutaneous melanoma-specific genomic signatures is needed to optimize targeted-therapeutic precision strategies.

Necroptosis is seen as a novel form of programmed necrotic cell death, which plays an important part in overcoming apoptosis resistance, triggering and amplifying antitumor immunity in cancer therapy, similar to apoptosis in mechanism and necrosis in morphology^4–6^. The necroptosis is regulated by distinct proteins, including RIPK1, RIPK3, and MLKL^7^. For example, RIPK1 has been associated with a variety of innate immune receptors and sensors. Downstream of these receptors and associated adaptors have been implicated in the regulation of multiple responses, including necroptosis, pyroptosis, and apoptosis^8^. In melanoma, selective inhibition of the RIPK3/MLKL axis by loss of RIPK3 is essential to prevent necroptosis^9^. Necroptosis is immunogenic and activates antitumor responses, potentially providing a therapeutic strategy to eliminate apoptosis-resistant tumor cells. Regulation of necroptosis-related proteins is often downregulated in cancer cells, suggesting that cancer cells may also escape necroptosis to survive, whereas the expression level of key mediators is elevated in certain tumors^6^. Necroptosis has been reported to have pro-metastatic and immunosuppressive effects, which may promote tumorigenesis and cancer metastasis^6^.

Melanin pigmentation regulates the expression of multiple genes and plays a critical role in many cellular processes in melanoma cells^10–12^. It can affect the behavior of melanoma cells, their surrounding environment, susceptibility to the therapy, and viability of immune cells^10,13,14^. Slominski et al.^11^ reported that induction of melanogenesis is associated with marked upregulation of HIF-1α and HIF-1-dependent pathways, resulting in increased aggressiveness of melanoma. Pigmentation levels affect the sensitivity of melanoma cells to chemotherapy and radiotherapy. The inhibition of melanogenesis makes pigmented melanoma more sensitive to cyclophosphamide and radiotherapy^13,15^. Janjetovic et al.^14^ revealed that a high pigmentation level was associated with melanoma resistance to hydroxyderivatives of vitamin D3. Cellular melanogenesis could affect the necroptosis of melanoma cells. Death induced by protein-binding polysaccharides (PBPs) is associated with induction of RIPK1/RIPK3/MLKL-mediated necroptosis^16,17^. In melanoma cells with active melanogenesis, induction of depigmentation with a melanin synthesis inhibitor restores melanoma cell sensitivity to RIPK1/RIPK3/MLKL-mediated necroptosis^18^.

Melanoma is one of the most immunogenic tumors and is most likely to respond well to immunotherapy^19^. It is an excellent model for evaluating innovative immunotherapies such as checkpoint inhibitors^19,20^. Despite significant advances in cancer immunotherapy, the majority of melanoma patients do not respond or relapse due to primary or acquired drug resistance, resulting in treatment failure^21^. The plasticity of melanoma cells leads to a phenomenon known as “immune escape”, whereby cancer cells acquire a less immunogenic phenotype and the ability to suppress anti-tumor immune cells within the tumor microenvironment (TME)^22,23^. Therefore, it is crucial to develop the necroptosis classification and melanoma-specific signature, which might guide the screening of melanoma patients with good responses to immunotherapy.

## Materials and methods

### Data extraction

The RNA-seq data and relevant clinicopathological data were obtained from The Cancer Genome Atlas Breast Cancer (TCGA-SKCM) and Genotype-Tissue Expression (GTEx) databases using University of California, Santa Cruz (UCSC) Xena Browser (https://xenabrowser.net/)^24^. Curated survival data and phenotypes were also obtained. In addition, mRNA expression and clinical data of an external validation set were retrieved from the Gene Expression Omnibus (GEO) database (ID: GSE65904). The samples with missing clinical information and overall survival (OS) < 30 days were excluded and 454 patients were included in the analysis. Moreover, Copy Number Variation (CNV) data was collected from the UCSC website, and the immunology treatment response data was from The Cancer Immunome Atlas (TCIA) and tumor immune dysfunction and exclusion (TIDE).

### Determination of differentially expressed genes (DEGs) associated with necroptosis

The 67 necroptosis-associated genes were obtained from Supplementary Materials of previous publication^25^, and then intersected with genes from GSE65904 dataset to obtain a total of 65 necroptosis-related genes (Table S1). The “limma” package was utilized for identifying DEGs between tumor and adjacent normal tissues with the following cutoff for adjustment: *P* < 0.05 and |Fold change|> 1.5.

### Consensus cluster for necroptosis genes

The “NMF” package in R software was used to perform non-negative matrix factorization (NMF) clustering analysis with expression matrix of differentially expressed necroptosis-related genes, and to identify different necroptosis phenotypes in cutaneous melanoma. The correlations between different necroptosis patterns and prognosis were analyzed.

### Immune cells infiltration analysis in TME

CIBERSORT algorithm was employed to evaluate the relative immune cells’ contents in the immune microenvironment [19]. The differences in immune cell infiltration in different necroptosis patterns were compared and analyzed.

### Generation of necroptosis signature

Necroptosis-associated genes were first screened for their prognostic values in the TCGA cohort by univariate Cox proportional hazards regression analysis. Then, LASSO Cox regression analysis was applied to narrowing down candidate genes using R package “glmnet”. Subsequently, the patients’ risk score was calculated based on the results of multivariate Cox regression analysis. Patients were ranked by their risk score and subsequently assigned into high- and low-risk groups. The median value of the risk score from the TCGA cohort was used as the cut-off point. Kaplan–Meier analysis with a two-sided log-rank test using “survminer” package was conducted to determine the prognostic value of the risk score. The time-dependent receiver operating characteristic (ROC) curve using “timeROC” R package was employed to assess the signature’s predictive accuracy, and the area under curve (AUC) metric was calculated. Principal component analyses (PCA) were carried out with “prcomp” in the “stats” R package based on the retrieved genes. Moreover, in the testing set, the same methods were adopted to examine the accuracy of the risk score model.

### Assessment of immune landscape in TME

The marker gene set consisting of 547 genes (Table S2) which represent 22 immune cell types was used to assess immune cell infiltration by the CIBERSORT algorithm^26^. Spearman correlation was performed between the risk score and the population of immune infiltrate estimates. The immune, stromal, and ESTIMATE scores for each sample were also analyzed.

### Prediction of the immune response

Expression of 47 immune checkpoints was compared between the two risk subgroups (Table S3). Immunophenoscore (IPS) for cutaneous melanoma patients was generated based on the expression of MHC molecules, immunomodulators, effector cells, and suppressor cells, and could robustly reflect the response to immunotherapy. IPS, ranging from 0 to 10, was obtained from TCIA (https://tcia.at/home)^27^. TIDE method was also used to predict the response of immunotherapy^28^. The response of each sample to anti-PD-1/PD-L1 and anti-CTLA4 immunotherapy was evaluated using TIDE algorithm according to the gene expression profiles. TIDE was developed on the basis of two primary mechanisms of tumor immune evasion: the induction of T cell dysfunction in tumors with high infiltration of cytotoxic T lymphocytes (CTLs) and the prevention of T cell infiltration in tumors with low CTL levels. The TIDE algorithm can determine the signatures of T cell dysfunction by testing how the expression of each gene in tumors interacts with the CTL infiltration level to influence patient survival and response to immunotherapy^28^.

### Analysis of pigmentation score among different risk groups

Gene set variation analysis (GSVA) enrichment analysis was performed based on the expression of 101 pigmentation-related genes, which were derived from MSigDB database (https://www.gsea-msigdb.org/gsea/index.jsp). The GSVA score is considered a pigmentation score, which represents the pigmentation status of each patient. Differences in pigmentation scores were compared between high and low-risk groups.

### Independent prognostic significance of signature and stratification analysis

Univariate and multivariate Cox proportional hazards regression analyses were conducted using “survival” package to evaluate the prognostic capacity of the risk score in distinguishing between low- and high-risk patients and for its independence from other pertinent clinicopathological parameters. These parameters were age, gender, tumor location, and pathological stage. In addition, the KM analysis was conducted to elucidate differences between two risk subgroups based on age (≤ 60 and > 60 years), tumor location (primary tumor, regional lymph node, regional cutaneous, and distant metastasis), stage (I-II and III-IV), ulceration (< = 1.5 and > 1.5), tumor status (tumor-free and with tumor).

### Drug sensitivity analysis

To estimate necroptosis-related prognostic model in the clinical treatment of cutaneous melanoma, we employed the “pRRophetic” package to calculate TCGA project of the cutaneous melanoma dataset for the IC50 of commonly used chemotherapeutic agents. The algorithm allows participants to apply baseline tumor gene expression profiles to predict clinical chemotherapy response, which is obtained by establishing statistical models from the gene expression and drug sensitivity data derived from cell lines in the Cancer Genome Project.

### Construction of a predictive nomogram

A nomogram was constructed based on the multivariate analysis using “rms” package to predict the 3- and 5-year survival probability. Time-dependent ROC curve was further drawn to evaluate the prognostic capacity of the nomogram-based prediction model for overall survival by AUC metric. In addition, calibration plots were also drawn to assess the predictive performance of the model.

### Functional analysis

To evaluate the possible biological functions of necroptosis-related genes, GSEA was performed to detect the associated GO terms and KEGG pathways between the low- and high-risk groups. The Hallmarks of C2 KEGG v.7.4 was selected, and the number of permutations was set to 1000 times. The outcomes that meet *P* < 0.05 were considered statistically significant.

## Results

### Genetic variation landscape of necroptosis genes in cutaneous melanoma

**Figure 1:**
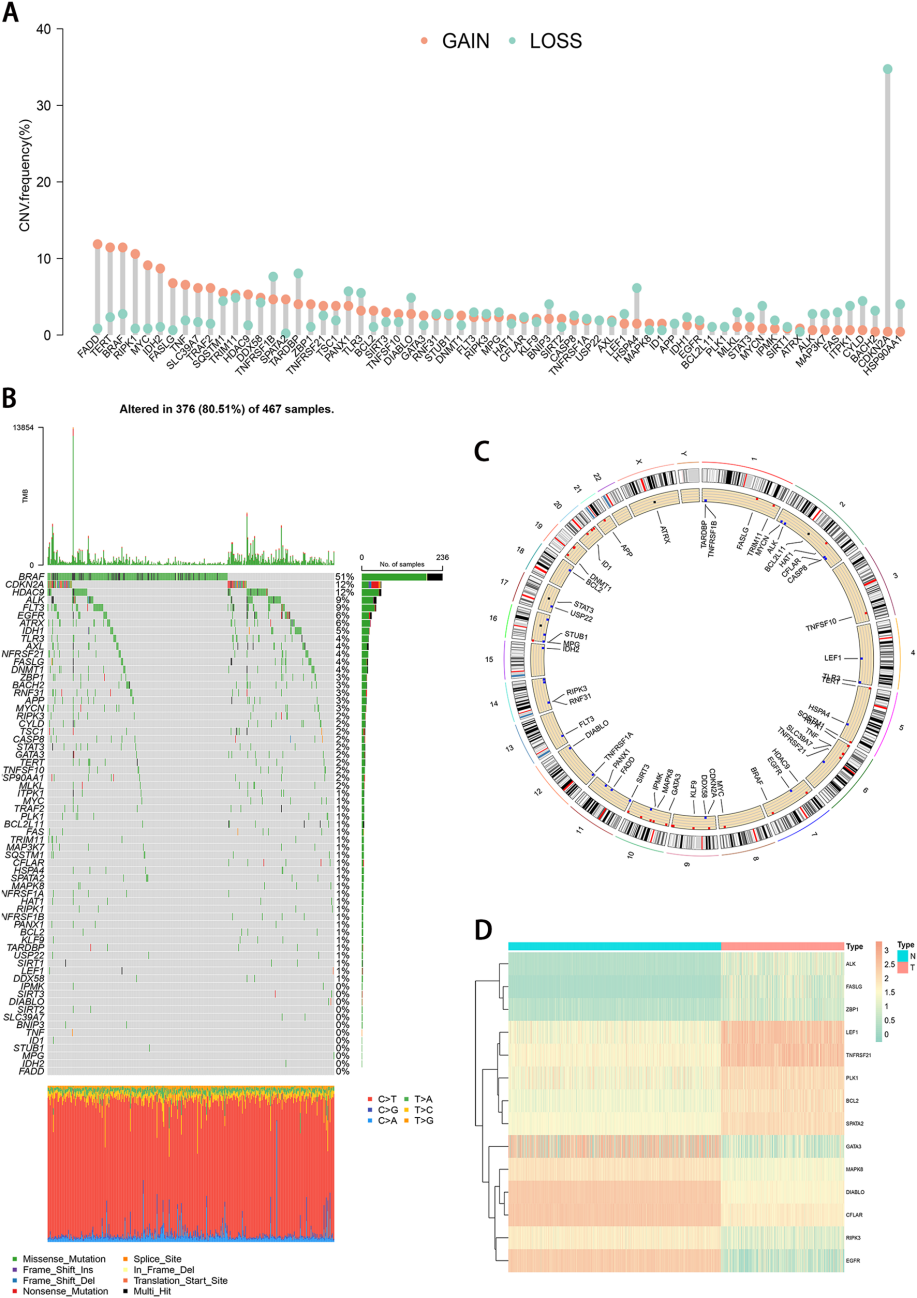
Landscape of genetic variation of 65 necroptosis-related genes in melanoma. (**A**) The CNV frequency of 65 necroptosis-related genes. (**B**) The somatic mutation of 65 necroptosis-related genes. (**C**) The location of the 65 necroptosis-related genes in human chromosomes. (**D**) Expression levels of 14 differentially expressed necroptosis-related genes between normal tissue and tumor tissues.

We explored the genetic variation of necroptosis-related genes in cutaneous melanoma from 3 aspects in the TCGA database: CNV, somatic mutations, and differential expression in the transcriptome. We found that a similar number of necroptosis-related genes gained copy number and CNV deletion (Fig. 1A). Genomic mutations were common in these genes with 376 (80.51%) of 467 patients having occurred genetic changes, in which BRAF (51%) had the most genetic alteration. CDKN2A and HDAC9 followed with 12% mutational frequency (Fig. 1B). The location of the 65 necroptosis-related genes in human chromosomes could be seen in Fig. 1C. The differential analysis in normal tissue and tumor tissue showed 14 necroptosis-related genes with significantly differential expression in cutaneous melanoma (Fig. 1D). The alteration and genetic variation of necroptosis-related genes act as an important part in regulating the happening, aggravation, and prognosis of cutaneous melanoma.

### DEGs between normal and tumor tissue specimens

The 65 necroptosis-associated genes were comparatively assessed for their expression in the pooled GTEx and TCGA data involving 813 normal and 473 tumor tissue specimens, and 14 DEGs were identified (*P* < 0.05). Of these DEGs, 6 (EGFR, GATA3, DIABLO, CFLAR, RIPK3, and MAPK8) were repressed, while (ZBP1, PLK1, SPATA2, BCL2, ALK, FASLG, TNFRSF21, and LEF1) were upregulated in cancer cases (Fig. 1D).

### Identification of necroptosis patterns based on DEGs

**Figure 2:**
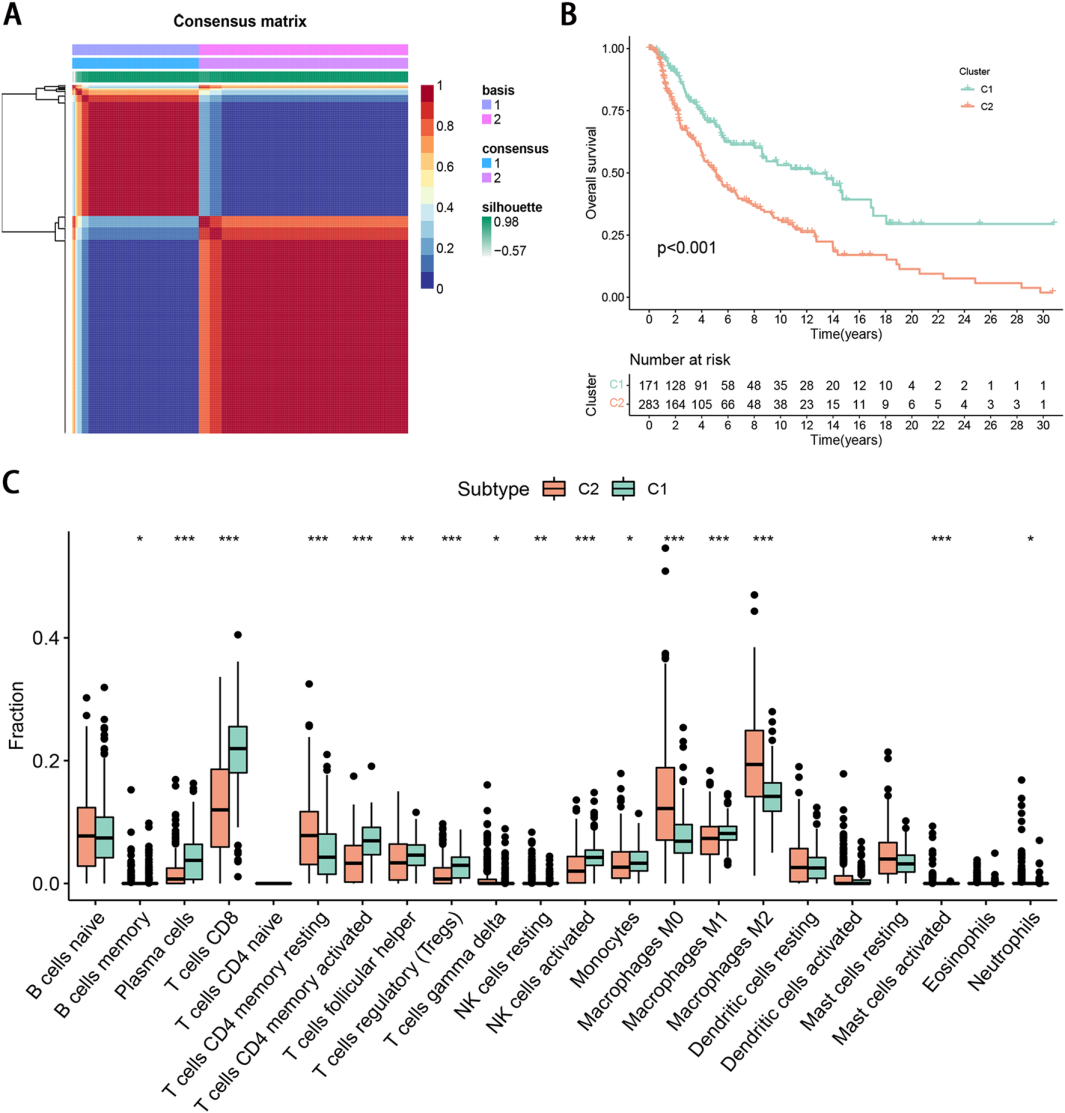
Identification of necroptosis patterns based on DEGs. (**A**) NMF consensus clustering for the k value was 2. (**B**) Kaplan–Meier curves of OS for patients with the two clusters. (**C**) The distinction in 22 kinds of immune infiltration cells between the two clusters.

Based on the expression profile of 14 DEGs, NMF consensus clustering analysis was carried out. At a clustering variable (k) of 2, the magnitude of the cophenetic correlation coefficient begins to fall, and intragroup and intergroup correlations were high and low, respectively, suggesting the SKCM cohort clustered in two groups (C1 and C2; Supplementary Fig. 1). We marked them necroptosis cluster A (n = 171) and B (n = 283), respectively (Fig. 2A; Table S4). The Kaplan–Meier curves showed that OS in the necroptosis cluster A was significantly better than that in the necroptosis cluster B (Fig. 2B).

### Immune cells infiltration analysis in TME

To understand the characteristics of immune infiltration cells in different clusters, we conducted the distinction of 22 kinds of immune cell infiltration between the two clusters. Among the immune infiltration cells, a majority of immune cells were found infiltrated into the necroptosis cluster A, including activated memory CD4 + T cells, CD8 + T cells, plasma cells, activated NK cells, monocytes, M1 macrophages, helper follicular T cells, and regulatory T cells (Tregs) (Fig. 2C).

### Prognostic gene-based model in TCGA cases and external validation of the risk signature

**Figure 3:**
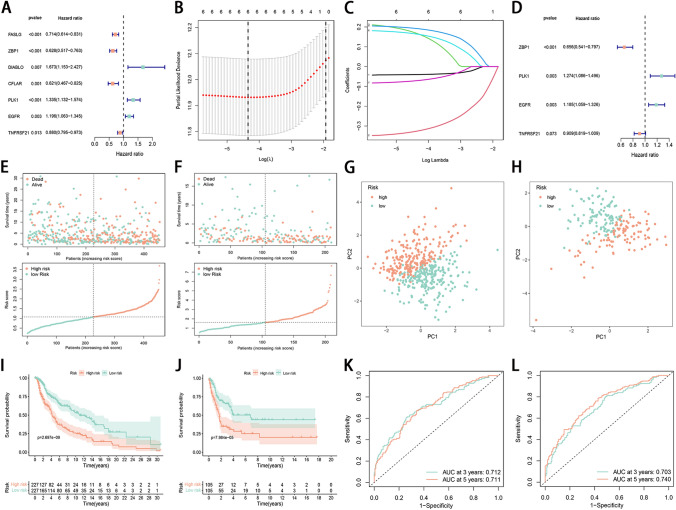
Necroptosis-related signature constructed and validated in the TCGA and GEO cohorts, repsectively. (**A**) The 7 necroptosis-related genes with *P* < 0.05 and their hazard ratios from univariate Cox proportional hazards regression analysis. (**B**) Tuning parameter selection in the LASSO model for OS-relevant genes in the TCGA cohort. (**C**) The LASSO coefficient profile of the 6 necroptosis-related genes in the TCGA cohort. The vertical line indicates the coefficient selected by LASSO. (**D**) Forrest plot showed that a total of 4 necroptosis-related genes were identified as prognosis-related by multivariate cox analysis. (**E** and **F**) The distribution and value of the risk scores in the TCGA and validation cohorts. (**G** and **H**) PCA of melanoma patients according to the risk score in the TCGA and validation cohorts. (**I** and **J**) Kaplan–Meier curves show that the low-risk group has significantly longer overall survival compared to the high-risk group in both the TCGA and validation cohorts. (**K** and **L**) ROC curve analysis shows the predictive efficiency of the established risk score in TCGA and validation cohorts.

Univariate Cox proportional hazards regression analysis showed that 7 out of the 14 necroptosis-related genes had *P* < 0.05 in the TCGA cohort (Fig. 3A). To eliminate collinearity of the variables and avoid overfitting of the prognostic model, these 7 genes were undergone the LASSO regression analysis in the TCGA dataset (Fig. 3B and C). Subsequently, 6 candidate genes were identified for further multivariate Cox regression analysis. Finally, the signature was constructed according to the expression levels of 4 genes (ZBP1, PLK1, EGFR, TNFRSF21, and ACAT2) (Fig. 3D). Risk score = -− 0.421 × (ZBP1 expression value) + 0.242 × (PLK1 expression value) + 0.169 × (EGFR expression value) -0.095 × (TNFRSF21 expression value). Based on the obtained median score, patients were divided into the low- and high-risk subgroups. In the TCGA and validation cohorts, high-risk patients had elevated mortality and reduced survival time in comparison with their low-risk counterparts (Fig. 3E and F). PCA showed good discriminative performance of the necroptosis signature in TCGA and validation cohorts (Fig. 3G and H). Kaplan–Meier survival analysis with a two-sided log-rank test in the TCGA and validation cohorts showed patients in the high-risk group had significantly shorter OS compared to the patients in the low-risk group (*P* < 0.05) (Fig. 3I). The prognostic value of the signature was further tested in the validation cohort whereby a similar significant difference in OS was observed between the low- and high-risk groups (*P* < 0.05) (Fig. 3J). The AUC for the 3-year (0.712) and 5-year (0.711) survival rates in the TCGA cohort and the 3-year (0.703) and 5-year (0.740) survival rates in the validation cohort showed favorable specificity and sensitivity of the signature in predicting OS (Fig. 3K and L).

### Correlation between risk score and TME

**Figure 4:**
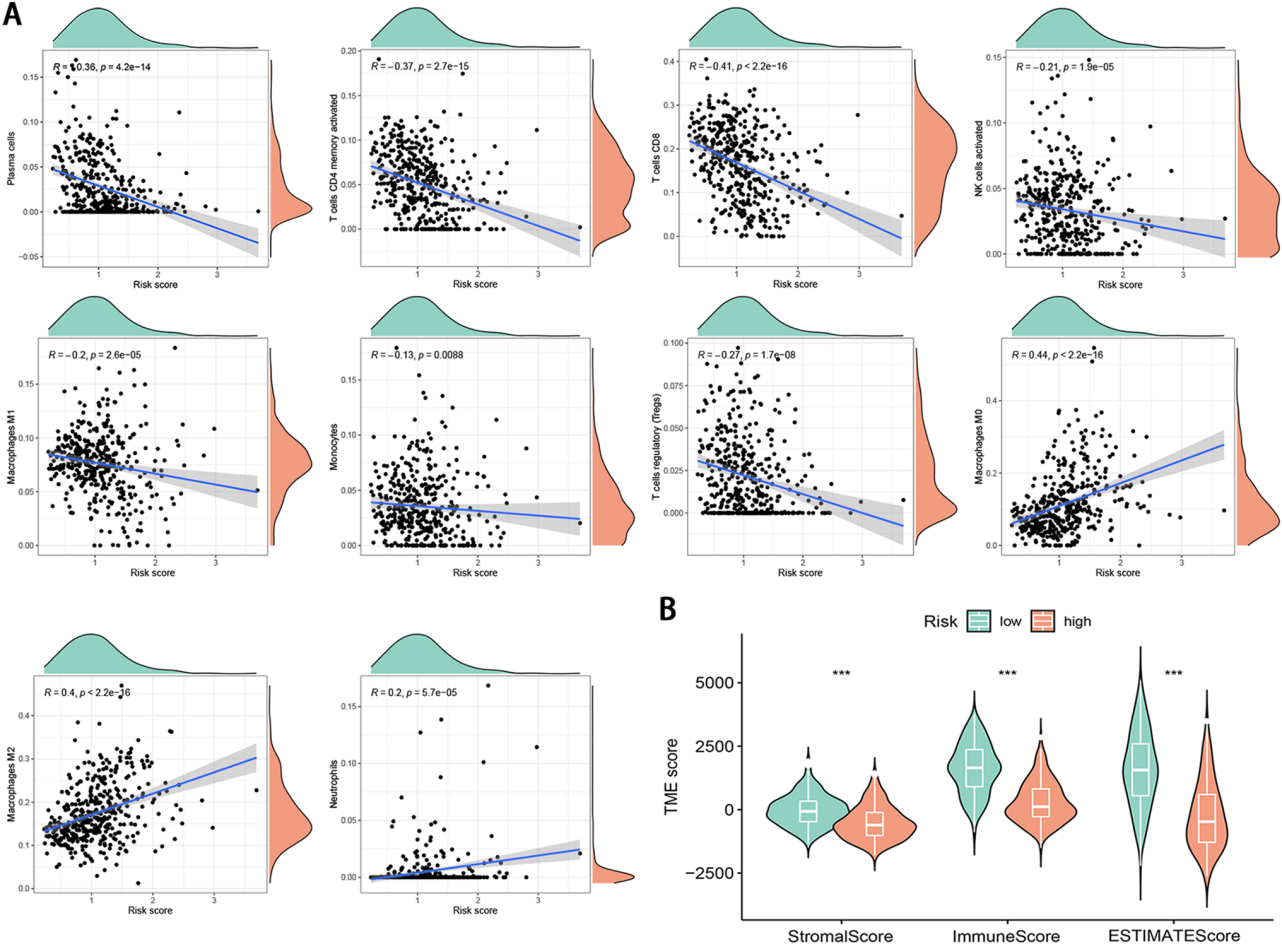
Immune landscape between low- and high-risk groups. (**A**) The infiltration levels of 22 immune cell types in the two risk groups. (**B**) The relationship between signature and the TME score.

We performed a CIBERSORT algorithm to evaluate the infiltrating level of immune cells in the tumor microenvironment and made comprehensive comparisons with the risk score. The high-risk score was negatively associated with infiltration of immune cells, including plasma cells, activated memory CD4 + T cells, CD8 + T cells, activated NK cells, M1 macrophages, regulatory T cells (Tregs), and memory B cells, and positively associated with infiltration of M0 macrophages, M2 macrophages, and neutrophils (Fig. 4A). Furthermore, we observed that the risk score had a strong negative correlation with the TME scores (Fig. 4B).

### Correlation between risk score and response to immunotherapy

**Figure 5:**
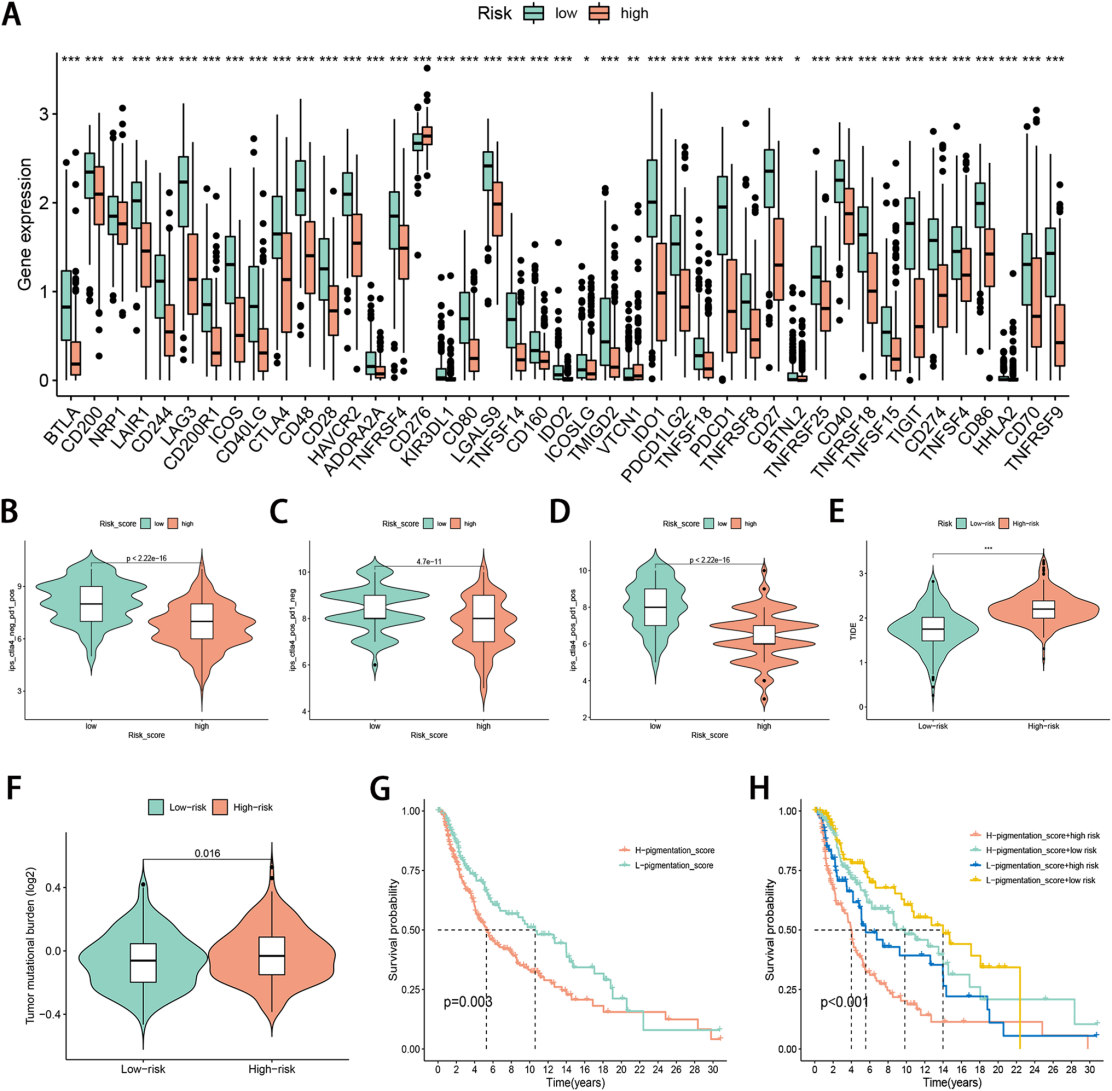
The relationships between risk score and immunotherapy. (**A**) Difference of immune checkpoint expression between high- and low-risk groups. (**B**–**D**) Difference of immunogenicity between high- and low-risk groups. (**E**) Difference of TIDE score between low- and high-risk groups. (**F**) Comparison of pigmentation score in the high- and low-risk groups. (**G**) Survival analysis of high and low pigmentation score group. (**H**) Survival analysis of melanoma stratified by pigmentation score and risk score.

We explored the relationships between risk score and response to immunotherapy and evaluated whether risk score could serve as a predictor to immune response. We selected 47 immune checkpoints to analyze their expression differences between the two risk subgroups and observed a higher expression of 41 immune checkpoints in patients with low-risk scores (Fig. 5A). In The Cancer Immunome Atlas, the IPS which was based on immunogenicity could achieve high accuracy in predicting the immunotherapy response of patients. Therefore, we analyzed the relationship of IPS between high and low-risk score groups. Interestingly, we found that the total IPS for PD-1 or CTLA-4, or both PD-1 or CTLA-4 blockers in the low-risk score group was significantly higher than that in the high score group, which meant that patients with lower risk scores tended to have a better response to immunotherapy (Fig. 5B–D). Next, the TIDEs were also calculated to predict response to immunotherapy. Similarly, patients with the low-risk score have a lower TIDE score (*P* < 0.05), suggesting a better response to immunotherapy (Fig. 5E). Taken together, the necroptosis signature established by us could serve as a robust predictor to immune response.

### Correlation of risk score with pigmentation score

We compared the pigmentation score of the high- and low-risk groups. The result indicated that the pigmentation score of the high-risk group was visibly higher than those of the low-risk group (Fig. 5F). Based on the median value of the patient’s pigmentation score, we divided the patients into low and high pigmentation score subgroups. KM survival analysis indicated that the OS rate in the high pigmentation score group was obviously lower than that in the low pigmentation score group (Fig. 5G). Stratified survival analysis demonstrated that the prognostic value of the risk score was not affected by the pigmentation score. Risk score subtypes showed significant differences in survival between low and high pigmentation score subgroups (Fig. 5H). Taken together, the risk score may be an effective indicator that is independent of the pigmentation score and can effectively evaluate the prognosis of patients.

### Independent prognostic significance of signature and stratification analysis

**Figure 6:**
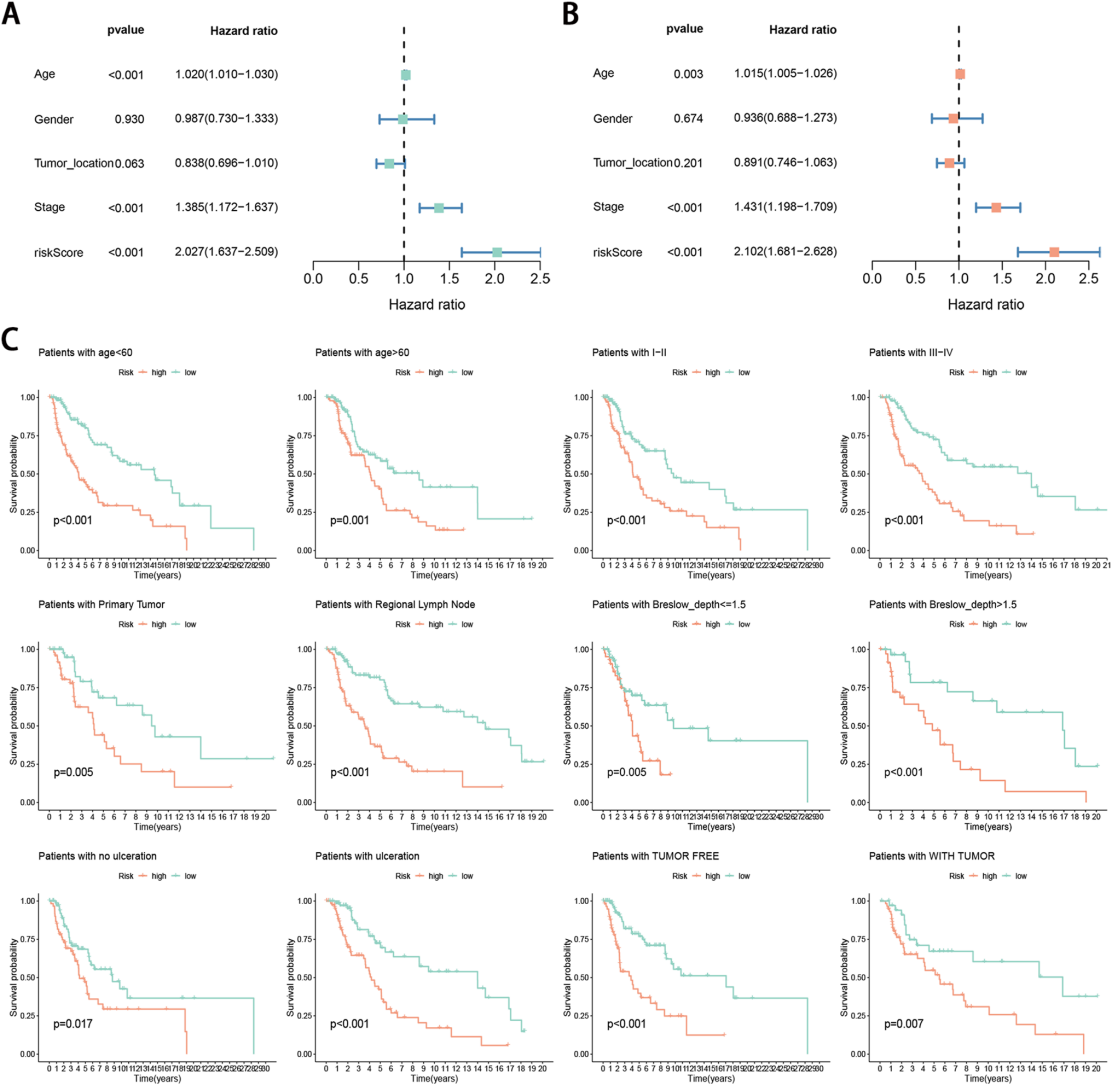
Univariate and multivariate Cox proportional hazards regression analysis for pertinent clinicopathological parameters. (**A**) Higher age, advanced stage, and high-risk groups were significantly poor prognostic factors in univariate analysis. (**B**) The high-risk group remains as an independent poor prognostic factor (*P* < 0.05) in the multivariate analysis. (**C**) Survival rates of the high- and low-risk patients in the subgroups based on clinicopathological characteristics.

Univariate analysis indicated that among the pertinent clinicopathological parameters, high age, advanced stage, and high-risk score were poor prognostic factors (Fig. 6A). In multivariable analysis, after adjustment for other confounders, age, stage, and the risk score were still independent prognostic indicators (Fig. 6B). To confirm the prognostic discriminatory power of the signature, we performed stratified survival analysis in various clinical subgroups, including age (age < 60 and age > 60), gender (female and male), tumor location (primary tumor, regional lymph node, and distant metastasis), stage (I-II and III-IV), Breslow depth (< = 1.5 and > 1.5), ulceration (no and yes), and tumor status (tumor-free and with tumor). As the result shown in Fig. 6C, the OS of the low-risk patients based on age (*P* < 0.001 in both age < 60 and age > 60), stage (*P* < 0.001 in both I-II and III-IV), tumor location (*P* = 0.005 in primary tumor; *P* < 0.001 in regional lymph node), Breslow depth (*P* = 0.005 in <  = 1.5, *P* < 0.001 in > 1.5), ulceration (*P* = 0.017 in no and *P* < 0.001 is yes), and tumor status (*P* < 0.001 in tumor-free and *P* = 0.007 in with tumor) was significantly higher than that of high-risk patients.

### Drug sensitivity analyses

We compared the sensitivity of two risk groups to common anticancer drugs to identify potential cutaneous melanoma treatment modalities. As shown in Supplementary Fig. 2, patients in the high-risk group were more sensitive to Docetaxel, Elesclomol, and Pazopanib, while those in the low-risk group were more sensitive to Gemcitabine, Gefitinib, Cyclopamine, Lenalidomide, and Metformin.

### Construction of prognostic nomogram

**Figure 7:**
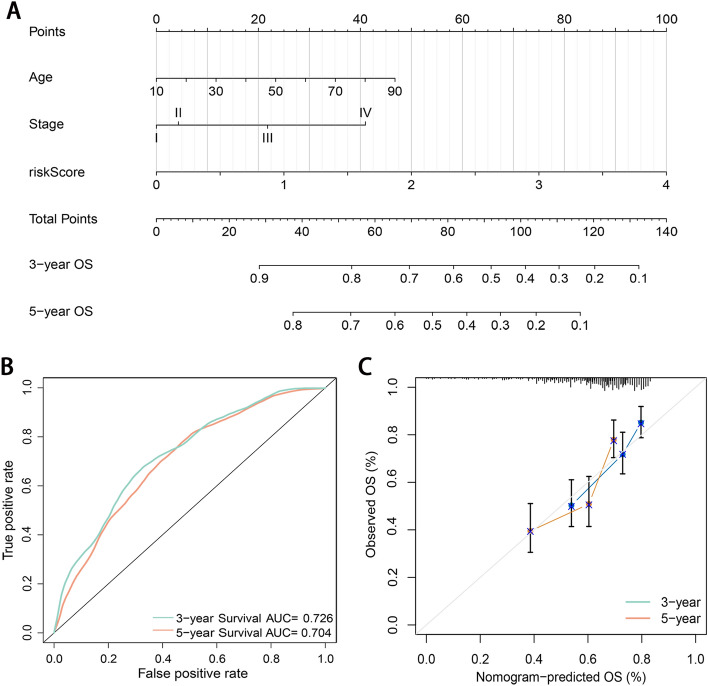
Construction and validation of the nomogram. (**A**) Nomogram for predicting 3-year and 5-year OS of melanoma patients incorporating independent prognostic factors from multivariate Cox proportional hazards regression. (**B**) AUCs of time-dependent ROC curves at 3- and 5-year OS for the independent prognostic factors. (**C**) Calibration curves corrected for deviations in agreement between the predicted and observed OS rates at 3 and 5 years.

To explore the potential value of the signature in clinical practice, we constructed a nomogram based on risk scores and clinical variables to predict the 3- and 5-year survival rates. A nomogram was constructed integrating the age, TNM stage, and risk score in the multivariate analysis (Fig. 7A). AUCs of time-dependent ROC curve at 3 and 5 year-OS for the nomogram-based prediction model were 0.726 and 0.704 (Fig. 7B). Calibration plots revealed that the nomogram showed perfect concordance between the observed and predicted survival rates at 3- and 5-year (Fig. 7C).

### Gene Set Enrichment Analysis (GSEA)

We conducted GSEA between the low- and high-risk subgroups using the entire gene network based on the TCGA dataset. We observed that several pathways with enrichment in the low-risk group were related to immunity with the filter criteria of FDR q value < 0.05 (Table S5), including chemokine signaling pathway, T cell receptor signaling pathway, Toll-like receptor signaling pathway, natural killer cell-mediated cytotoxicity, and B cell receptor signaling pathway (Supplementary Fig. 3).

## Discussion

Melanoma is one of the most immunogenic tumors because it has an extremely high genomic mutational load and has the highest potential to elicit specific adaptive antitumor immune responses^19^. The necroptosis is regulated by distinct proteins, including RIPK1, RIPK3, and MLKL, and is characterized to be inhibited by the necrostatin-1^7^. Necroptosis acts as an integral part of the induction and amplification of cancer immunity^4^. RIPK3 is required to regulate cytokine expression in DCs which is a key sentinel in regulating immune homeostasis^29^. Necroptosis initiates adaptive immune responses via releasing DAMPs into the tissue microenvironment. Tumor cells undergoing necroptosis can trigger robust antitumor immunity in *vivo* and in *vitro*, and immune checkpoint inhibitors (ICIs) can synergistically enhance their efficacy, even in ICI-resistant tumors^4^. Hence, necroptosis induction may provide promising therapeutic prospects, especially for patients who underwent drug resistance to traditional chemotherapy or immunotherapy. Although lots of studies focused on a single necroptosis biomarker or interaction between a certain sort of immune cells and necroptosis genes, few researchers investigated the landscape of genetic variation of necroptosis genes and TME infiltration characteristics modulated by necroptosis signature.

In the present study, we first revealed CNV frequency, somatic mutation frequency, chromosomal locations, and expression level of 65 necroptosis-related genes, and found that there was no significant correlation between the CNV and transcriptional expression. We identified 14 differently expressed necroptosis-related genes between tumor and normal tissues. After that, we made an NMF consensus cluster analysis and identified 2 necroptosis clusters based on 14 DEGs. Compared with cluster B, necroptosis cluster A had better OS, and the subsequent TME analyses showed that the contents of infiltrated immune cells in cluster A were significantly higher than that in cluster B. Next, the combined analysis of Cox and LASSO regression was applied to establish a necroptosis signature. The prognostic risk signature showed great predictive ability both in the TCGA and validation datasets. We also explored the risk score and infiltrated immune cells and TME risk scores. We found the low-risk group could be described as immune activated type, while the high-risk group an immune failure type. Furthermore, we found that low-risk patients exhibited higher levels of immune checkpoints and showed higher IPS and lower TIDE scores, suggesting a better response to immunotherapy. Similarly, analyses of drug response demonstrated significant differences between the two risk subgroups. To quantify patient survival and enhance the use of the necroptosis signature, we built a nomogram integrating age, tumor stage, and risk score. Functional enrichment analysis indicated that gene sets associated with the low-risk group were significantly enriched in immune signaling pathways.

TME is a crucial mediator of cancer progression and therapeutic outcome. The TME status correlates with patient response to immunotherapy in multiple cancers^30^. Thus, explaining the diversity and complexity of the TME is an integral step in enhancing the predictive power and clinical guidance of immunotherapy. Previous studies have shown crosstalk between cells undergoing necroptosis and the remodeling of the immune microenvironment^4^. Notably, we revealed a correlation between two necroptosis clusters and the immune microenvironment, which may guide distinct therapeutic approaches in the risk groups. Furthermore, the low-risk group tends to have significantly higher infiltrating levels of the most immune cell types than the high-risk group. Increasing evidence shows that T cells play an important role in the anti-cancer immune response^31–33^. This corresponds to our result that patients in the high-risk group tended to have a lower level of CD4 + and CD8 + T cells. The result indicated that patients of higher risk tend to have an unfavorable tumor-infiltrating lymphocytes pattern. Moreover, the signature was also significantly associated with innate immune cell types, including macrophages, monocytes, and NK cells. Since immune checkpoint blockade targeting PD-1, PD-L1 and CTLA4 has shown promising anti-tumor effects by reversing the immunosuppressive effects of tumors, the expression of immune checkpoints has attracted widespread attention as a biomarker for identifying melanoma patients undergoing immunotherapy^34–36^. In our research, the necroptosis signature obtained has good performance in predicting immune checkpoint treatment. The expression levels of most immune checkpoints were observed significant up-regulation in the low-risk subgroup. Hence, ICIs that target these checkpoints may be an optimal therapeutic approach for these patients. In addition, we found that low-risk patients showed higher IPS and lower TIDE scores, suggesting a better response to immunotherapy. Finally, we found that multiple chemotherapeutic agents have better sensitivity for high and low-risk groups, respectively, so that suitable chemotherapeutic agents can be selected according to different risk groups.

The three genes in the signature had been reported to be involved in tumorigenesis and progression in cutaneous melanoma, which enhances the predictive performance of the signature. ZBP1 is a Zα domain-containing protein that recognizes viral and endogenous Z-RNA^37^. ZBP1 recognizes Z-RNA in macrophages to activate the NLRP3 inflammasome and PANoptosis, an inflammatory programmed cell death pathway regulated by the PANoptosome complex with pyroptosis, apoptosis, and/or necroptosis key features of apoptosis^38^. Growing evidence suggests that ZBP1-mediated necroptosis is involved in antitumor immunity and tumorigenesis^39^. Yang et al.^39^ revealed that ZBP1-MLKL necroptotic signaling potentiated radiation-induced antitumor immunity via intratumoral STING pathway activation. In a breast cancer study, ZBP1 expression is dramatically upregulated in necrotic tumors. Knockdown of ZBP1 blocked tumor necroptosis during tumor development and inhibited metastasis^40^. Polo-like kinase-1 (PLK1) is one of the key regulators of cell cycle progression. PLK1 had been reported to play crucial roles in the metabolism and progression of cutaneous melanoma^41–43^. Schmit et al.^44^ revealed that PLK1 expression was elevated in both melanoma tissue specimens and melanoma cells compared to normal skin tissue and melanoma cells. Knockdown of PLK1 resulted in significantly reduced viability and growth of melanoma cells. In addition, knockdown of PLK1 resulted in decreased clonal survival, cell cycle arrest, and apoptosis in melanoma cells. Su et al.^42^ reported that higher PLK1 levels were associated with worse OS and disease-free survival (DFS), which is consistent with our results. In addition, Deeraksa et al.^45^ demonstrated that PLK1 expression is elevated in androgen-insensitive prostate cancer (PCa) cells and silence of PLK1 promotes necroptosis-induced cell death in LNCaP-AI cells. Therefore, it is promising to exploit necroptosis through PLK1 inhibition for the treatment of castration-resistant PCa. EGFR is a member of the RTK family and upregulated EGFR promotes migration and invasion of melanoma cells. The viability of melanoma cells receiving EGFR inhibitor was reduced, suggesting that EGFR is a promising target for anticancer therapy^46^.

Melanogenesis is the most characteristic feature of melanoma^10^. Cellular melanogenesis could affect the regulated cell death of melanoma cells. Pawlikowska et al.^16,17^ revealed that melanin content in melanoma cells can affect the induction of RIPK1/RIPK3/MLKL-mediated necroptosis by Coriolus Versicolor fungus-derived protein-PBPs. In melanoma cells with active melanogenesis, induction of depigmentation with a melanin synthesis inhibitor restores melanoma cell sensitivity to RIPK1/RIPK3/MLKL-mediated necroptosis^18^. These findings are consistent with our findings. In this study, the pigmentation score of the high-risk group was visibly higher than those of the low-risk group. In addition, melanogenesis was associated with clinical outcomes of melanoma patients^10^. Brożyna et al.^47^ indicated that patients with melanotic melanomas have the significantly shorter OS and DFS than those with amelanotic lesions. In our study, we also found that the OS rate in the high pigmentation score group was obviously lower than that in the low pigmentation score group, consistent with a previously published article.

## Conclusions

Our study revealed the extensive effect of necroptosis signature on cutaneous melanoma microenvironment, and succeed in identifying two distinctive necroptosis phenotypes and establishing signature, which closely correlated with response to immunology in cutaneous melanoma. Our results provide insight for improving personalized cancer immunotherapy.

## Supplementary Information

Supplementary Figures.

Supplementary Tables.

## Data Availability

The datasets generated and/or analysed during the current study are available in the TCGA-SKCM (https://portal.gdc.cancer.gov/) and GEO (https://www.ncbi.nlm.nih.gov/geo/; ID: GSE65904).

## Abbreviations

NMF: Non-negative matrix factorization
TME: Tumor microenvironmental
OS: Overall survival
IPS: Immunophenoscore
TIDE: Tumor Immune Dysfunction and Exclusion
TCGA: The Cancer Genome Atlas Breast Cancer
GEO: Gene Expression Omnibus
CNV: Copy Number Variation
ROC: Receiver operating characteristic
AUC: Area under curve
PCA: Principal component analysis
TCIA: The Cancer Immunome Atlas
GSEA: Gene Set Enrichment Analysis
ICIs: Immune checkpoint inhibitors
