# Supplementary material for: Necroptosis-associated classification combined with tumor microenvironment characteristic analysis of cutaneous melanoma
Source: Sci Rep. 2022 May 24;12:8752. doi: 10.1038/s41598-022-12676-6 (PMC9130269; doi:10.1038/s41598-022-12676-6)
Supplement: Supplementary file 1 — Supplementary Figures. [file 41598_2022_12676_MOESM1_ESM.docx]

**Supplementary Figures**

**
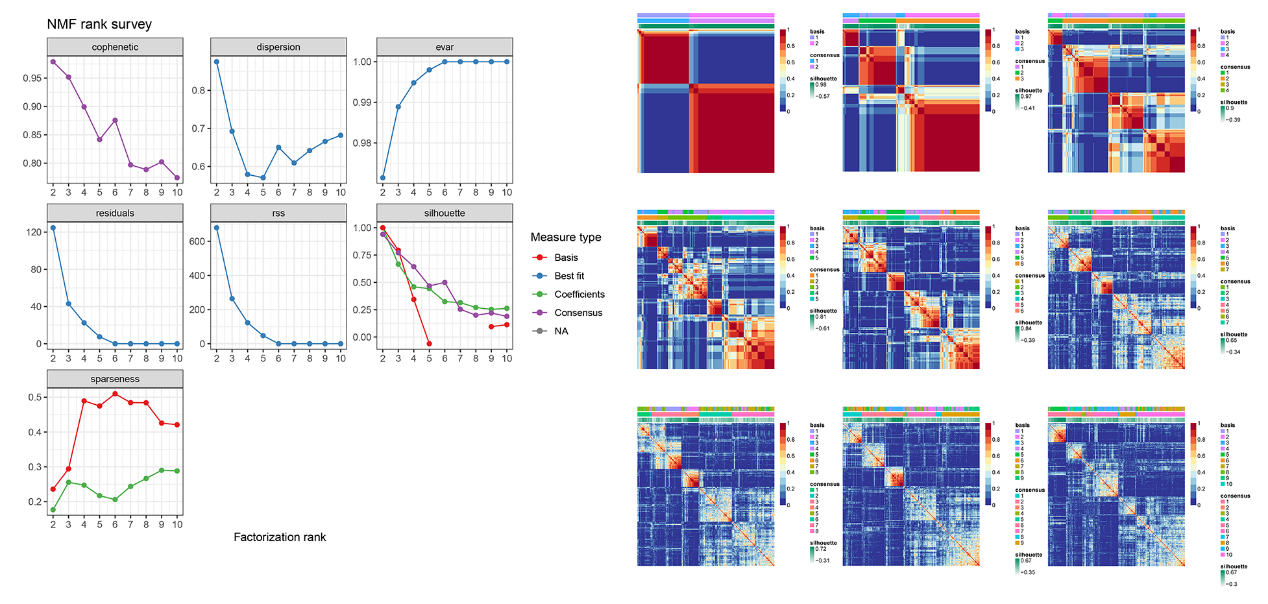
**

**Supplementary Figure 1** Heatmap of NFM consensus cluster analysis and cophenetic correlation coefficients for k = 2-10.


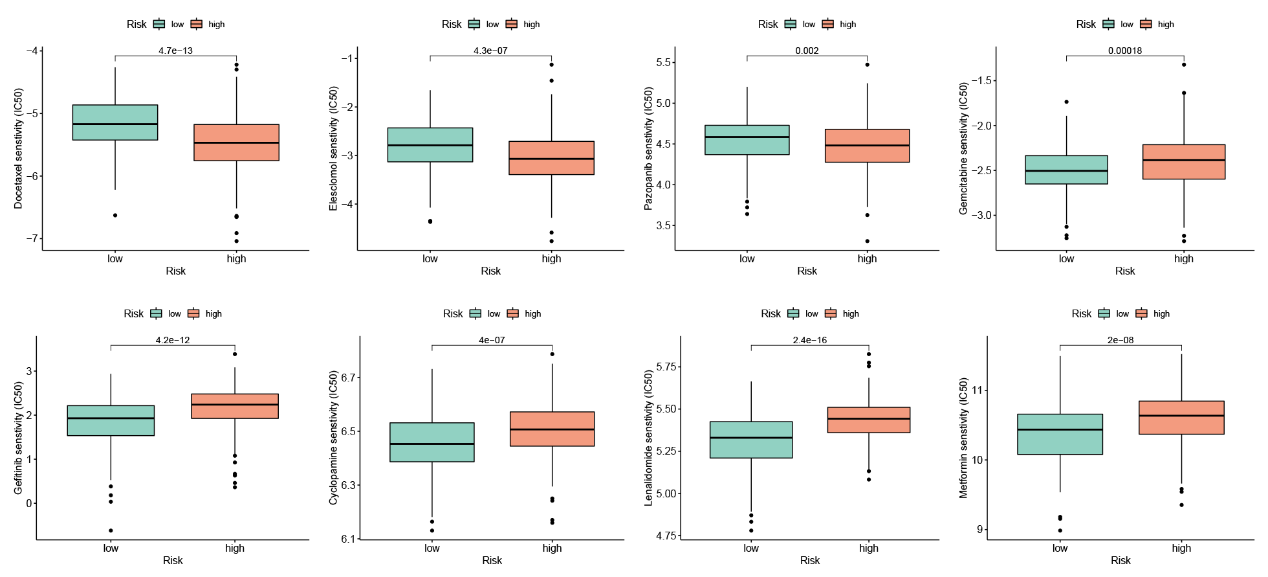


**Supplementary Figure 2** Drug sensitivity analyses between high- and low-risk groups. The IC50 values of four chemotherapeutic drugs in the high- and low-risk groups.


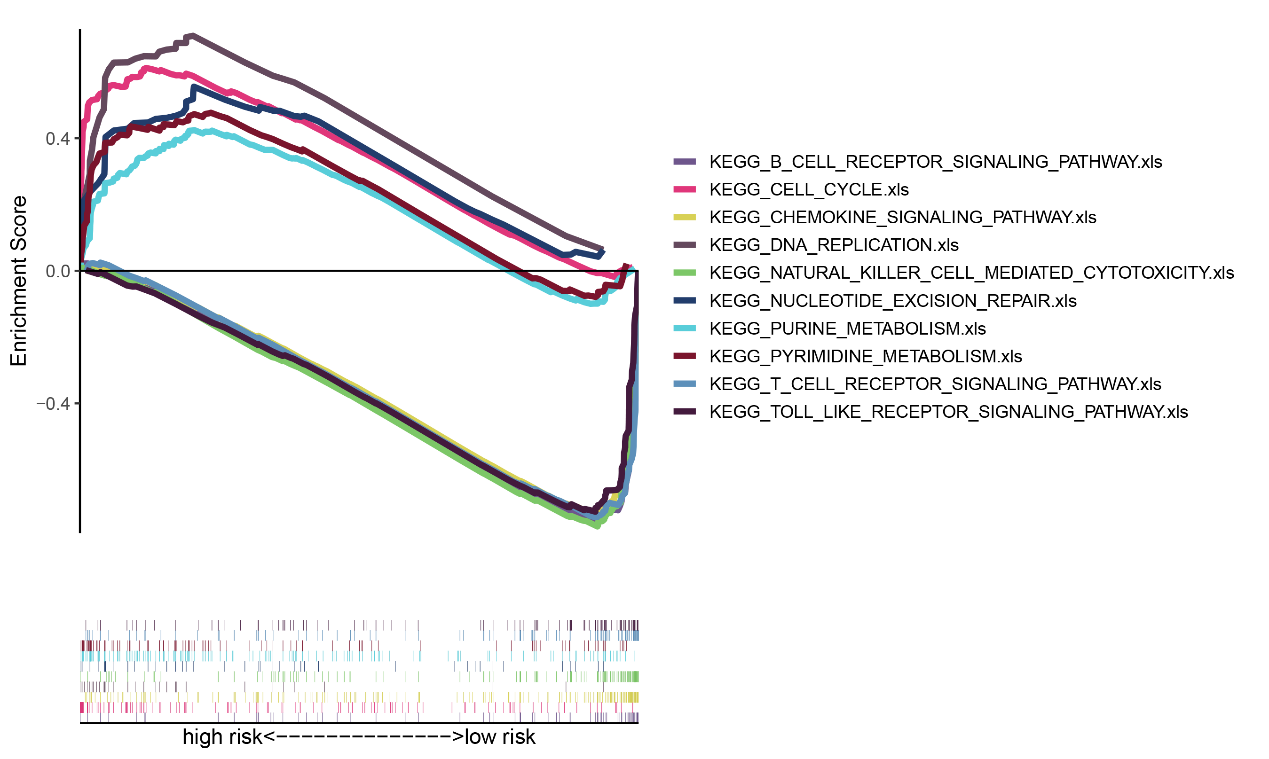


**Supplementary Figure 3** Gene Set Enrichment Analysis between high- and low-risk sets.
